# Nitric Oxide Influences HSV-1-Induced Neuroinflammation

**DOI:** 10.1155/2019/2302835

**Published:** 2019-02-11

**Authors:** Joanna Cymerys, Andrzej Kowalczyk, Katarzyna Mikołajewicz, Anna Słońska, Małgorzata Krzyżowska

**Affiliations:** ^1^Department of Preclinical Sciences, Faculty of Veterinary Medicine, Warsaw University of Life Sciences, Ciszewskiego 8, 02-786 Warsaw, Poland; ^2^PORT Polish Center for Technology Development, Stablowicka 147, 54-066 Wroclaw, Poland

## Abstract

Herpes simplex virus type 1 (HSV-1) has the ability to replicate in neurons and glial cells and to produce encephalitis leading to neurodegeneration. Accumulated evidence suggests that nitric oxide (NO) is a key molecule in the pathogenesis of neurotropic virus infections. NO can exert both cytoprotective as well as cytotoxic effects in the central nervous system (CNS) depending on its concentration, time course exposure, and site of action. In this study, we used an *in vitro* model of HSV-1-infected primary neuronal and mixed glial cultures as well as an intranasal model of HSV-1 in BALB/c mice to elucidate the role of NO and nonapoptotic Fas signalling in neuroinflammation and neurodegeneration. We found that low, nontoxic concentration of NO decreased HSV-1 replication in neuronal cultures together with production of IFN-alpha and proinflammatory chemokines. However, in HSV-1-infected glial cultures, low concentrations of NO supported virus replication and production of IFN-alpha and proinflammatory chemokines. HSV-1-infected microglia downregulated Fas expression and upregulated its ligand, FasL. Fas signalling led to production of proinflammatory cytokines and chemokines as well as induced iNOS in uninfected bystander glial cells. On the contrary, NO reduced production of IFN-alpha and CXCL10 through nonapoptotic Fas signalling in HSV-1-infected neuronal cultures. Here, we also observed colocalization of NO production with the accumulation of *β*-amyloid peptide in HSV-1-infected neurons both in vitro and in vivo. Low levels of the NO donor increased accumulation of *β*-amyloid in uninfected primary neuronal cultures, while the NO inhibitor decreased its accumulation in HSV-1-infected neuronal cultures. This study shows for the first time the existence of a link between NO and Fas signalling during HSV-1-induced neuroinflammation and neurodegeneration.

## 1. Introduction

Herpes simplex virus type 1 (HSV-1) causes a contagious infection that affects approximately 60% to 95% of adults worldwide. HSV-1 is associated mainly with infections of the mouth, pharynx, face, eye, and central nervous system (CNS). The virus persists in the body by becoming latent in the cell bodies of nerves after the primary infection. People infected with HSV-1 can expect to have several (typically four or five) outbreaks (symptomatic recurrences) within a year. HSV-1 has the ability to replicate in neurons and glial cells and to produce acute focal, necrotizing encephalitis localized in the temporal and subfrontal regions of the brain [1, 2]. Herpes simplex encephalitis (HSE) predominantly affects children and the elderly, is one of the most common forms of viral encephalitis, and has remarkably poor outcomes despite the availability of good antiviral therapy [3–5].

Pathogen-induced neurodegeneration occurs by both direct effects on brain cells and indirect inflammatory and oxidative effects. Mounting evidence suggests that HSV-1 exposure to neuronal cells results in cellular production of *β*-amyloid proteins (A*β*). Mouse brains infected with HSV-1 showed increases in A*β*42 five days postintranasal infection compared to uninfected controls [6]. In HSV-1-infected human neuroblastoma cells, experimentally induced oxidative stress was found to significantly enhance the accumulation of intracellular A*β* and to inhibit A*β* secretion [7]. HSV-1 interactions with oxidative stress are significant because oxidative damage is thought to occur early in the pathogenesis of Alzheimer disease (AD) [8].

Microglia and astroglia are consistently found surrounding amyloid plaques in AD brains [9]. A*β* deposition causes a microglial-mediated inflammatory response [10]. Proinflammatory molecules have been shown to be involved in pathways of neuronal apoptosis [11]. A*β*-stimulated microglia secrete TNF-*α* and glutamate in vitro, resulting in simultaneous activation of neuronal TNF-*α* and N-methyl-D-aspartate (NMDA) receptors and subsequent neuronal apoptosis [11]. Additional neurotoxic compounds produced by activated microglia include superoxide, hydrogen peroxide, and nitric oxide. Fas and other receptors from the tumor necrosis factor (TNF) receptor family upon interaction with their ligands (e.g., FasL) trigger the so-called death receptor pathway of apoptosis [12]. Fas is not expressed in the adult brain under physiological conditions, but it has been detected in the brains of patients with AD, in human malignant astrocytic brain tumors, during ischemic injury, in multiple sclerosis (MS), and in HIV encephalopathy (HIVE) [13, 14], while FasL expression during neuroinflammation is detected mainly on infiltrating myeloid cells or on the activated microglia [15, 16].

Nitric oxide (NO) is a signalling molecule synthesized from the amino acid L-arginine via enzymes called NO-synthases (NOS) [16]. There are three different kinds of NOS [16]. NOS is induced in a variety of experimental virus infections in rats and mice, including neuroviruses, such as Borna disease virus, herpes simplex virus type 1, and rabies virus [17–19]. Viral or synthetic dsRNA, also in conjunction with interferon gamma (IFN-*γ*), increases the expression and activity of NF-*κ*B, which further induces iNOS expression [20].

Previous studies have shown that HSV-1 is susceptible to the effects of NO *in vivo* in mice and rats [19]. Despite its antiviral activity, NO is not always beneficial, as it can promote the pathogenesis of HSV-1 by damaging cells in host tissues [19]. In a prooxidant environment, NO reacts with superoxide anion to generate peroxynitrite (ONOO−), a highly reactive anion [21, 22]. Peroxynitrite has been shown to induce lipid peroxidation, as well as functional alterations to proteins through tyrosine nitration (nitrotyrosination) [21, 22]. These modifications are molecular markers of AD [21, 22].

It was suggested that increased expression of all NOS forms in astrocytes and neurons contributes to the synthesis of peroxynitrite which leads to generation of nitrotyrosine, which can be detected in blood and cerebrospinal fluid (CSF) of AD patients [21]. Also, aberrant expression of nNOS in cortical pyramidal cells colocalized with nitrotyrosine in the brains of AD patients and it correlated with the cognitive impairment [21, 22].

We have previously shown that the lack of the Fas-dependent pathway of apoptosis plays an important role in the elimination of the inflammation surrounding the HSV-2-infected sites and regulation of monocyte-induced inflammation during HSV infection [23]. Here, we hypothesize that both the NO and Fas/FasL pathways are involved in HSV-1 induced neuroinflammation and neurodegeneration during HSV-1 infection. The Fas/FasL pathway leads to increased levels of NO observed during both *in vitro* and *in vivo* HSV-1 infection, which in turn can contribute to A*β* aggregation.

## 2. Materials and Methods

### 2.1. Cell Lines and Virus

Murine astrocyte C8-D1A and African green monkey kidney (Vero) cell lines were purchased from the American Type Culture Collection (ATCC® CRL-2541™ and ATCC® CCL-81™, respectively). C8-D1A cells were grown in Dulbecco's modified essential medium (D-MEM), supplemented with 10% fetal bovine serum (FBS), 4 mM L-glutamine, 1 mM sodium pyruvate (Gibco by Thermo Fisher Scientific, Carlsbad, CA, USA), 5 g/l glucose, 100 U/ml penicillin, 100 *μ*g/ml streptomycin, and 0.25 *μ*g/ml amphotericin B (Thermo Fisher Scientific) in standard conditions. Vero cells were grown in Eagle's minimum essential medium, supplemented with 10% FBS, 100 U/ml penicillin, 100 *μ*g/ml streptomycin, and 0.25 *μ*g/ml amphotericin B (Thermo Fisher Scientific).

HSV-1 strain McKrae [24] was grown (PFU/ml) in African green monkey kidney (Vero) cells. Virus titers were determined by plaque assay on Vero cells.

### 2.2. Primary Neuronal Cultures and Mixed Glial Cultures

BALB/c mice were used to establish primary culture of murine neurons, as described before [25]. Neuronal cells were cultured in B-27 Neuron Plating Medium consisting of neurobasal medium, B-27 supplement, glutamine (200 mM), glutamate (10 mM), antibiotics (penicillin and streptomycin), 10% FBS, and horse serum (Thermo Fisher Scientific) in standard conditions. At day 3, 1 *μ*M AraC (cytosine *β*-D-arabinofuranoside) for 24 hrs was added. Four days after plating, the medium was removed and replaced with Neuron Feeding Medium (B-27 Neuron Plating Medium without glutamate). In this medium, murine neurons were maintained for the next 6 days, prior to treatment.

Mixed glial cultures were obtained as described by Draheim et al. [26]. In brief, whole brains of neonatal BALB/c mice were taken and blood vessel and meninges were carefully removed. Then, the whole brains of five mice were pooled together and digested with 0.25% trypsin/Hanks' balanced salt solution for 10 minutes. Next, the homogenate was filtered through a 70 *μ*m cell strainer (BD Biosciences) into a 50 ml conical tube. After rinsing the filter with PBS, the resulting cell suspension was centrifuged again at 300 g for 5 min at room temperature. The pellet was dissolved in Dulbecco's modified Eagle's/F12 medium with GlutaMAX (DMEM/F12) supplemented with 10% FBS, 100 units/ml penicillin, 100 *μ*g/ml streptomycin (Thermo Fisher Scientific), and 5 ng/ml murine recombinant granulocyte and macrophage colony stimulating factor (GM-CSF) (Sigma-Aldrich, St. Luis, MO, USA). Medium was changed every 72 h, and the mixed glial cells were used for experiments after two weeks of culture. The cultures consisted of 40% CD11b+ cells and 60% GFAP+ cells as assessed by flow cytometry (details below).

Both neuronal and mixed glial cultures were infected with HSV-1 at MOI = 1 for 24 h and subjected to treatment with the NO donor—sodium nitroprusside (SNP) (1000, 500, 100, 50, and 10 *μ*M)—and the inhibitor of iNOS—aminoguanidine (AMG, 50 *μ*M).

### 2.3. Intranasal HSV-1 Infection

Six- to 10-week-old male mice were anesthetized with isoflurane (Geulincx), and 10^6^ PFU (plaque-forming units) of the purified HSV-1 contained in 10 *μ*l was inhaled by the mice. The control mice inhaled PBS. The mouse colonies and all of the experimental procedures were performed according to the institutional animal care and use guidelines. From 24 h before infection up to 5 days of infection, mice received aminoguanidine sulphate (100 mg/kg body weight, intraperitoneally) (Sigma-Aldrich) dissolved in physiological saline. Five days later, mice of both treated groups were sacrificed and their trigeminal ganglia as well as brains were collected for further assays. The brains were fixed in 4% paraformaldehyde (PFA) in PBS, then saturated with 30% sucrose, frozen in liquid nitrogen, and used to prepare cryostat sections.

### 2.4. Virus Titration

Total DNA was isolated from trigeminal ganglia and brains preserved in RNA later (Thermo Fisher Scientific, MA, USA) using RNA/DNA Extracol kit (EURx, Gdansk, Poland). HSV-1 was detected using a HSV-1 probe labeled with FAM in a real-time PCR instrument Stratagene MX4000 Real-Time qPCR System (Agilent Technologies, USA) as described by Namvar et al. [27] and Orłowski et al. [28]. A plasmid vector pCR 2.1 containing an envelope glycoprotein (gB) gene fragment was constructed and purified by the Institute of Biochemistry and Biophysics Polish Academy of Sciences (Warsaw, Poland). Standard curve analysis was based on Ct values and serial of 10-fold dilutions of the plasmid standard with an initial concentration of 2.62 × 10^6^ HSV-1 genome copy numbers per reaction. A standard curve was included in each PCR run. The amplification efficiency (*E*) was calculated from the standard curves, using the formula *E* = 10(−1/*a*) − 1, where *a* is the slope. Data are expressed as the HSV-1 copy number per ng of the total DNA in the tissue.

### 2.5. Flow Cytometry Analysis

Cell suspensions prepared from cell cultures by the use of trypsin were pretreated with the Fc receptor block rat anti-CD16/32 antibody (2.4G2) (BD Biosciences, Franklin Lakes, NJ, USA) according to the manufacturer's protocol. Astrocytes were detected by anti-GFAP-FITC or APC-conjugated antibody (GA5, eBioscience), and microglia were stained with rat anti-CD11b-APC (M1/70, BD Biosciences). For detection of Fas and FasL, cells were washed in 1% FBS/PBS, and then, FITC-conjugated hamster anti-mouse Fas antibody (Jo2, BD Biosciences) and PE-conjugated hamster anti-mouse FasL antibody were used (MFL3, BD Biosciences). For all staining, rat IgG2a, rat IgG2b, and hamster IgG1 isotype antibodies conjugated with appropriate fluorochromes were used (BD Biosciences). Apoptosis in single cell suspensions was detected using Annexin V- APC Apoptosis detection kit (BD Biosciences), according to the manufacturer's protocol. The annexin V+ and propidium iodide (-) cells were scored as apoptotic, while annexin V+/propidium iodide (+) cells as secondary necrotic and annexin V (-)/propidium iodide (+) cells as necrotic. Apoptotic/secondary-, apoptotic/necrotic CD11b+-, or GFAP+-positive cells were detected by prestaining with anti-CD11b-FITC (M1/70, BD Biosciences) and anti-GFAP-FITC (GA5, eBioscience) antibodies. Intracellular antigens were detected using Cytofix/Cytoperm fixation/permeabilization kit (BD Biosciences) according to the manufacturer's protocol and by using anti-iNOS-PE antibody (CXNFT, eBioscience). The stained cell suspensions were analyzed in FACS Calibur for the percentage of positively stained cells or the mean fluorescence intensity.

### 2.6. Immunofluorescent Staining and Microscopy Analysis

Primary murine neurons or mixed glial cultures seeded on glass coverslips in a 6-well plate were infected with HSV-1. At 24 h p.i. (hour post infection), neuronal cells were washed twice in PBS (Sigma-Aldrich) and fixed in 3.7% paraformaldehyde/PBS (Sigma-Aldrich) for 10 min at room temperature (RT), and then, permeabilization with 0.5% Triton X-100 (Sigma-Aldrich) solution in PBS was performed. Before staining, slides with cell cultures or brain cryosections were blocked with PBS containing 1% bovine serum albumin (BSA) and 0.5% saponin (Sigma-Aldrich) for 30 min at RT. For immunophenotypic characterization and identification of iNOS, Fas, FasL, and A*β*, slides were stained by means of immunofluorescence with appropriate antibodies: anti-CD11b-APC (M1/70, BD Biosciences) and anti-GFAP-APC (GA5, eBioscience), anti-Fas-APC (Jo2, BD Biosciences), biotinylated anti-FasL (MFL3, BD Biosciences), anti-iNOS-PE (CXNFT, eBioscience), anti-NeuN-biotin (A60, Sigma), and anti-*β*-Amyloid (NAB228; Thermo Fisher Scientific) antibodies (dilution 1 : 100, overnight). After several washes with PBS, slides were incubated with secondary antibodies: Alexa 647 goat anti-mouse IgG (dilution 1 : 1000, 1 h, RT) and Streptavidin-Alexa 647 (dilution 1 : 200, 1 h, RT). The presence of viral antigen was detected by using rabbit mAb anti-HSV (dilution 1 : 250, 1 h, RT) and anti-rabbit FITC (dilution 1 : 200, 1 h, 37°C). Slides were mounted in ProLong Gold Antifade Reagent (Thermo Fisher Scientific) with DAPI. Noninfected cell cultures or uninfected brains served as negative control. Images were acquired using Leica SP8 resonant scanning confocal system (Leica Microsystem, Wetzlar, Germany). Stacks of confocal 8-bit images with a pixel size of 0.186 *μ*m and a 0.5 *μ*m Z step were acquired using 40x oil immersion objective (NA 1.30).

### 2.7. Western Blot Analysis

Cultured neuronal cells prepared as described before were first washed with ice-cold PBS and lysed in N-PER Neuronal Protein Extraction Reagent (Thermo Fisher Scientific) containing protease and phosphatase inhibitors (Halt Phosphatase Inhibitor Cocktail and Halt Protease Inhibitor Cocktail; Thermo Fisher Scientific), for 20 min on ice. The lysates were clarified by centrifugation for 15 min at 4°C. Quantitation of the protein content in lysates was performed with Micro BCA Protein Assay Kit (Thermo Fisher Scientific) and spectrophotometry on an Epoch BioTek spectrophotometer. Samples containing 20 *μ*g of protein were incubated with Laemmli sample buffer containing *β*-ME (Bio-Rad; Hercules, CA, USA) for 5 min at 95°C. Subsequently, the samples and protein markers were electrophoresed on a 10% polyacrylamide Bis-Tris Plus gel with MES running buffer and transferred onto a PVDF membrane. The membrane was blocked with 5% BSA in TBST and incubated overnight with primary mAb A*β* (NAB228; Thermo Fisher Scientific). After several washes in 0.1% Tris-buffered saline (TBS) Tween 20, blots were incubated with HRP-conjugated secondary antibodies for 1 h at RT and developed using enhanced chemiluminescence (Clarity Western ECL Substrate; Bio-Rad). The protein bands were visualized by ChemiDoc™ MP Imaging System (Bio-Rad).

### 2.8. Quantitative Reverse Transcriptase-Polymerase Chain Reaction for Cytokines and Chemokines

Total RNA was isolated from cells using Universal RNA Purification Kit (Eurx). Transcripts of IFN-*α*, CXCL9, CXCL10, TNF-*α*, and GADPH were quantified using TaqMan® Gene Expression Assays (Thermo Fisher Scientific). All PCR reactions were carried out with QuantiFast Probe RT-PCR Kit (QIAGEN, Hilden, Germany) using a real-time PCR instrument Stratagene MX4000 Real-Time qPCR System (Agilent Technologies) according to the manufacturer's protocol. The 2^−*∆∆*Ct^ method was used in calculating the relative ratio to control uninfected tissue.

### 2.9. Statistical Methods

Data are presented as the mean ± standard error of the mean (SEM) from at least three independent experiments. Data were analyzed using a two-tailed paired Student's *t*-test (normal distribution) or with nonparametric Kruskal-Wallis, and Wilcoxon tests were applied using BioStat 2009 software. In every analysis, values of *p* ≤ 0.05 were considered significant.

## 3. Results

### 3.1. Influence of NO upon HSV-1 Replication Depends on the Cell and Organ Type

**Figure 1:**
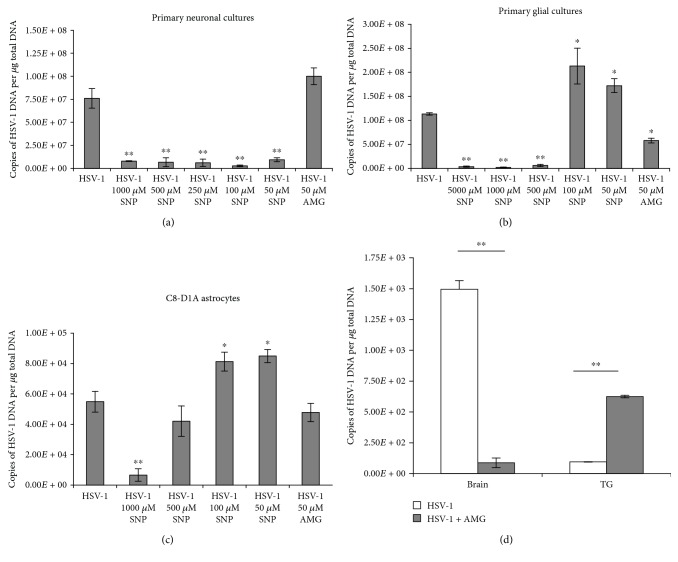
Nitric oxide influences HSV-1 replication. (a) Primary neuronal cultures and (b) primary glial cultures and (c) C8-D1A astrocyte cell line were subjected to treatment with the NO donor (SNP) or the inhibitor of iNOS (AMG) for 24 h and harvested for determination of HSV-1 DNA copies by PCR. (d) Numbers of HSV-1 DNA copies in the trigeminal ganglia (TG) and brains obtained at day 5 post intranasal infection with HSV-1 with or without treatment with AMG (100 mg/kg) were assessed by PCR. Means are expressed as mean ± SEM for *n* = 7; ^∗^significant differences with *p* ≤ 0.05, while ^∗∗^*p* ≤ 0.01 in comparison to infected controls.

To ascertain the susceptibility of HSV-1 replication in cell cultures to NO, sodium nitroprusside (SNP) was used as an exogenous NO donor at the concentration range of 1000-50 *μ*M and aminoguanidine sulphate (AMG) at the concentration of 50 *μ*M was used as an inhibitor of inducible nitric oxide synthase (iNOS). The SNP concentration ≤ 100 *μ*M and AMG concentration ≤ 50 *μ*M have been shown in the literature as nontoxic to neuronal cultures, while for glial cells, toxic doses of SNP are ≥1000 *μ*M [29, 30]. As indicated in Figure 1(a), the whole range of SNP concentrations inhibited HSV-1 replication in primary neuron cultures, while inhibition of iNOS caused no significant upregulation of HSV-1 replication (*p* ≤ 0.01) (Figure 1(a)).

The primary glial culture consisting of microglia and astrocytes showed a dose-dependent effect of NO upon HSV-1 replication (Figure 1(b)). Concentrations of SNP ≥ 500 *μ*M caused a significant inhibition of HSV-1 replication (*p* ≤ 0.01) (Figure 1(b)), while SNP concentrations ≤ 100 *μ*M significantly stimulated HSV-1 replication (*p* ≤ 0.05) (Figure 1(b)). The astrocyte C8-D1A cell line showed a similar effect: SNP at ≤100 *μ*M led to the significant upregulation of HSV-1 replication (*p* ≤ 0.05) (Figure 1(c)), while 1000 *μ*M SNP inhibited HSV-1 replication (*p* = 0.045). AMG had no effect upon HSV-1 replication in C8-1A astrocytes, while it significantly inhibited HSV-1 replication in mixed glial cells (*p* = 0.02) (Figure 1(b)). Furthermore, AMG showed different effects upon HSV-1 replication in the mouse model of neuroinfection (Figure 1(d)). At day 5 post infection (p.i.), HSV-1 DNA copies in mice treated with AMG were significantly higher in trigeminal ganglia (TG) in comparison to those in untreated mice (*p* ≤ 0.01) (Figure 1(d)). However, treatment with AMG led to significantly lower HSV-1 titers in the whole brain tissue (*p* ≤ 0.01) (Figure 1(d)).

### 3.2. Nitric Oxide Protects Infected Microglial Cells from Cell Death

**Figure 2:**
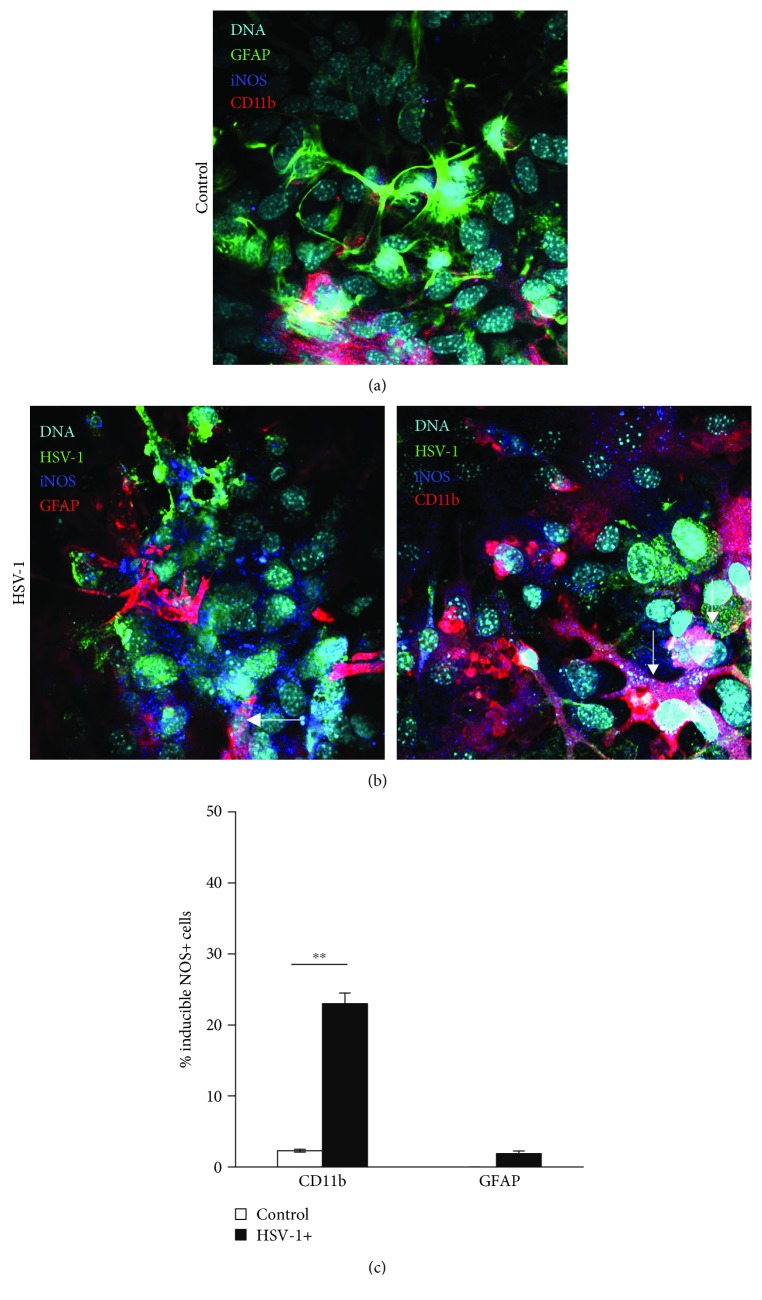
Cells positive for iNOS in HSV-1-infected mixed cultures. Representative confocal images of mixed glial cultures uninfected (a) or HSV-1 infected (b) at 24 h p.i. and stained for HSV-1, astrocytes (GFAP+), microglia (CD11b+), and iNOS (inducible NOS). Nuclei were counterstained with DAPI. (c) Percentage of CD11b+ and GFAP+ cells positive for iNOS accessed by flow cytometry in HSV-1-infected and control uninfected mixed glial cultures. Means are expressed as mean ± SEM for *n* = 3; ^∗^significant differences with *p* ≤ 0.05, while ^∗∗^*p* ≤ 0.01.

**Figure 3:**
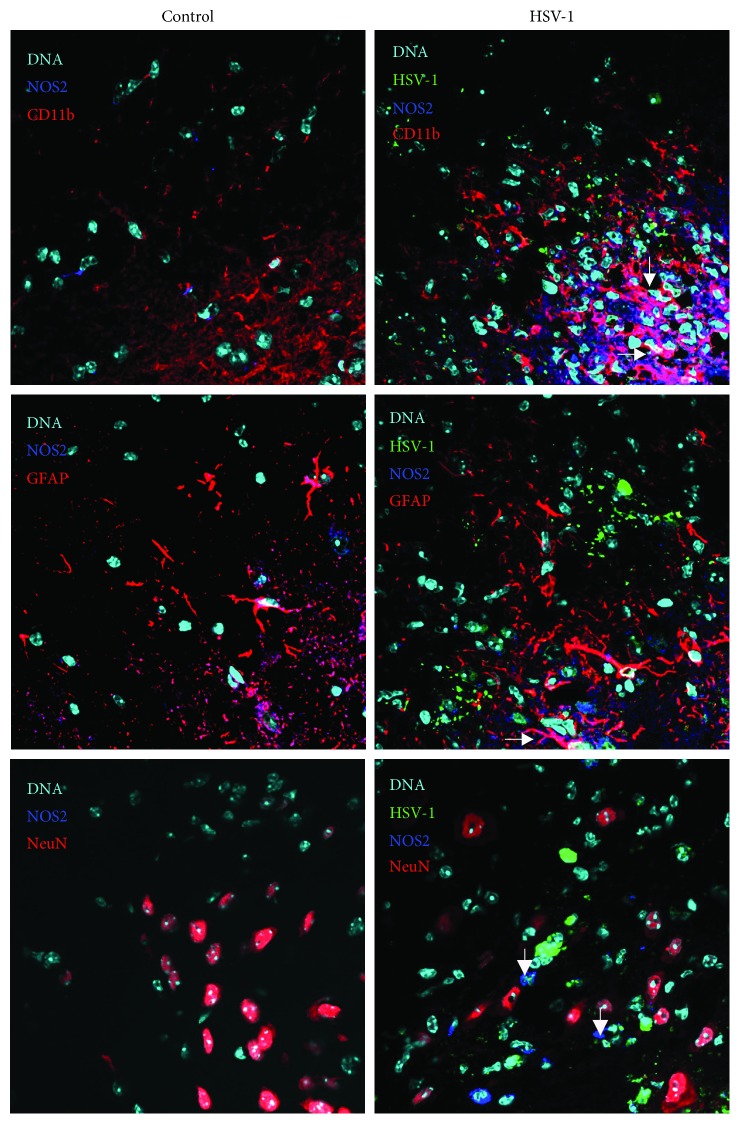
Microglia are the main source of iNOS in HSV-1-infected brains. Representative confocal images of brain stems obtained at day 5 p.i. with HSV-1. Neurons were identified as NeuN+-positive cells, while astrocytes as GFAP+ and microglia as CD11b+ and costained for iNOS and HSV-1. Nuclei were counterstained with DAPI.

The main sources of NO in HSV-1-infected primary neuronal and glial cell cultures were microglial cells and only a small proportion of astrocytes (Figures 2(a) and 2(b)). Flow cytometric analysis of mixed glial cultures for the percentage of iNOS+ cells showed that 23 ± 1.5% of CD11b+ were positive for iNOS, while 1.9 ± 0.54% of GFAP+ astrocyte cells were positive for iNOS (Figure 2(c)). In the brains of HSV-1-infected mice, HSV-1-positive cells were detected in the cerebellum, brain stem, midbrain, and tissue lining the lateral ventricles. Not only CD11b+-positive cells surrounding HSV-1-infected sites were positive for iNOS (Figure 3) but also single GFAP+ astrocytes positive for iNOS were found (Figure 3). The increased staining for iNOS was not associated with HSV-1 staining or staining for neurons (NeuN+) (Figure 3).

**Figure 4:**
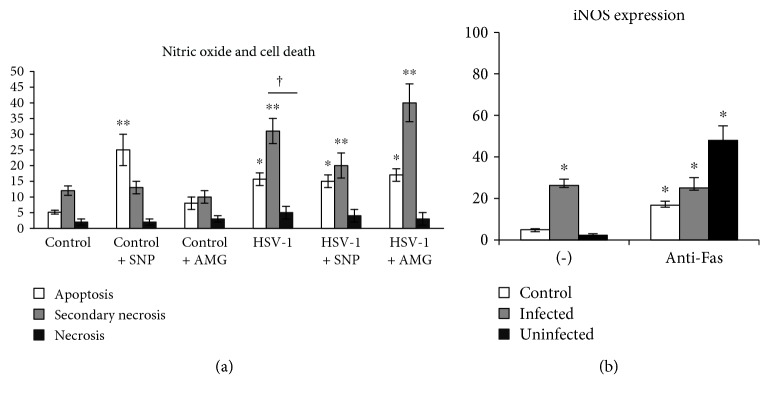
Nitric oxide protects microglia from cell death, while Fas signalling stimulates iNOS expression. (a) Percentage of apoptotic (annexin V+/propidium iodide -), secondary necrotic (annexin V+/propidium iodide+), and necrotic (annexin V-/propidium iodide+) CD11b+ cells in mixed glial cultures subjected to HSV-1 infection and treatment with the NO donor (SNP, 100 *μ*M) and the inhibitor of iNOS (AMG, 50 *μ*M) for 24 h. (b) Percentage of iNOS+ cells in mixed glial cultures infected with HSV-1 for 24 h. Means are expressed as mean ± SEM for *n* = 3; ^∗^significant differences with *p* ≤ 0.05, while ^∗∗^*p* ≤ 0.01 in comparison to uninfected control, † represents a significant difference with *p* ≤ 0.05 in comparison to HSV-1-infected cells.

Infected neuronal cultures showed no apoptosis upon HSV-1 infection (data not shown), while primary glial cells, consisting of astrocytes and microglia, showed upregulation of apoptosis and secondary necrosis (Figure 4(a)). Upon HSV-1 infection, only CD11b+ microglial cells show significant increase in apoptosis and secondary necrosis induction (*p* ≤ 0.05) (Figure 4(a)). Interestingly, while the nitric oxide donor caused increase in apoptosis induction in control, uninfected cells (*p* ≤ 0.05) (Figure 4(a)), it protected HSV-1-infected microglial cells from secondary necrosis (*p* ≤ 0.05) (Figure 4(a)). Both infected and uninfected neuronal cultures are not susceptible to Fas-induced apoptosis by cytotoxic recombinant sFasL (data not shown). Similarly, uninfected and infected mixed glial cultures also did not respond by apoptosis to stimulation of Fas receptor by sFasL (data not shown). Fas stimulation led to significant upregulation of iNOS expression not only in HSV-1-infected glial cells but also in uninfected cells from infected and control mixed glial cultures (Figure 4(b)).

### 3.3. Nitric Oxide Influences Production of Cytokines and Chemokines

To determine how NO can influence local inflammation during HSV-1, we measured expression levels of selected cytokines and chemokines in primary neuronal and mixed glial cultures at 24 p.i. We found that while no expression of cytokines or chemokines was detected in uninfected neuronal cultures, HSV-1 infection upregulated mRNA expression levels for IFN-alpha, TNF-alpha, CXCL9, and CXCL10 (Table 1). The NO donor—SNP—significantly downregulated expression of IFN-alpha, TNF-alpha, and CXCL10 in HSV-1-infected neuronal culture (*p* ≤ 0.05) (Table 1). Addition of sFasL to uninfected neuronal cultures led to upregulation of TNF-alpha and CXCL10 expression (Table 1). Furthermore, sFasL significantly induced expression of CXCL10 and reduced expression of TNF-alpha in HSV-1-infected cultures (*p* ≤ 0.05), while when coadded with the NO donor, it downregulated TNF-alpha and CXCL10 expression and upregulated CXCL9 expression (*p* ≤ 0.05) (Table 1). Upon HSV-1 infection, mixed glial cultures upregulated INF-alpha, TNF-alpha, CXCL9, and CXCL10 mRNA expression levels (*p* ≤ 0.01) (Table 2). The NO donor significantly upregulated mRNA expression levels for TNF-alpha, while it downregulated mRNA expression levels for IFN-alpha, CXCL9, and CXCL10 in HSV-1-infected mixed glial cultures (*p* ≤ 0.05) (Table 2). AMG significantly upregulated INF-alpha expression in comparison to HSV-1-infected cultures (*p* = 0.002) (Table 2). Addition of sFasL in uninfected mixed glial cultures led to upregulation of TNF-alpha, IFN-alpha, and CXCL9 expression levels (*p* ≤ 0.05) (Table 2).

However, sFasL downregulated TNF-alpha expression in HSV-1-infected glial cultures (*p* = 0.45) (Table 2). On the contrary, sFasL significantly upregulated CXC9 and CXCL10 expression levels in HSV-1-infected glial cultures (*p* ≤ 0.05) (Table 2). Coaddition of sFasL and the NO donor significantly downregulated TNF-alpha, CXCL9, and CXCL10 mRNA expression levels (*p* ≤ 0.05) (Table 2).

### 3.4. Fas and FasL Expression Can Be Regulated by Nitric Oxide

**Figure 5:**
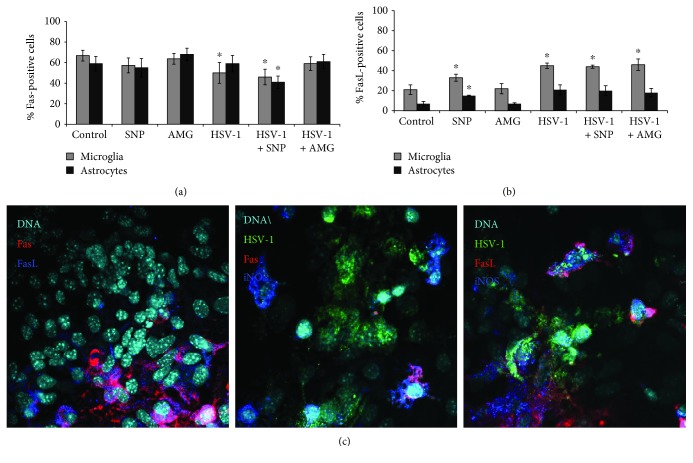
Nitric oxide regulates Fas/FasL expression on glial cells. Percentage of Fas-positive (a) and FasL-positive (b) microglial (CD11b+) and astrocyte (GFAP+) cells in mixed glial cultures subjected to HSV-1 infection and treatment with the NO donor (SNP 100 *μ*M) and the inhibitor of iNOS (AMG 50 *μ*M) for 24 h. (c) Representative confocal images of mixed glial cultures infected with HSV-1 for 24 h and stained for Fas, FasL, iNOS, and HSV-1. Means are expressed as mean ± SEM for *n* = 3; ^∗^significant differences with *p* ≤ 0.05, while ^∗∗^*p* ≤ 0.01 in comparison to uninfected control.

HSV-1 infection of mixed glial primary cultures led to significant downregulation of Fas expression on microglial cells (*p* ≤ 0.05) (Figure 5(a)) in comparison to uninfected cells, while it had no influence upon Fas expression on astrocytes (*p* ≥ 0.05) (Figure 5(a)). The NO donor—SNP—significantly downregulated Fas expression both on infected astrocytes and microglial cells (*p* ≤ 0.05) (Figure 5(a)) in comparison to uninfected control (*p* ≤ 0.05) (Figure 5(a)). AMG added to HSV-1-infected primary glial cultures upregulated Fas expression to the levels observed in control, uninfected cells (Figure 5(a)). SNP significantly increased FasL expression on microglial and astrocyte cells from uninfected primary cell cultures (*p* ≤ 0.05) (Figure 5(b)). HSV-1 infection led to significant upregulation of FasL expression only on microglia, while either SNP or AMG had no influence upon FasL expression in HSV-1-infected mixed glial cultures (Figure 5(b)). In HSV-1-infected mixed glial cultures, FasL-positive cells were identified as surrounding the infected cells, but also on infected cells. No Fas expression was detected on infected glial cells (Figure 5(c)).

**Figure 6:**
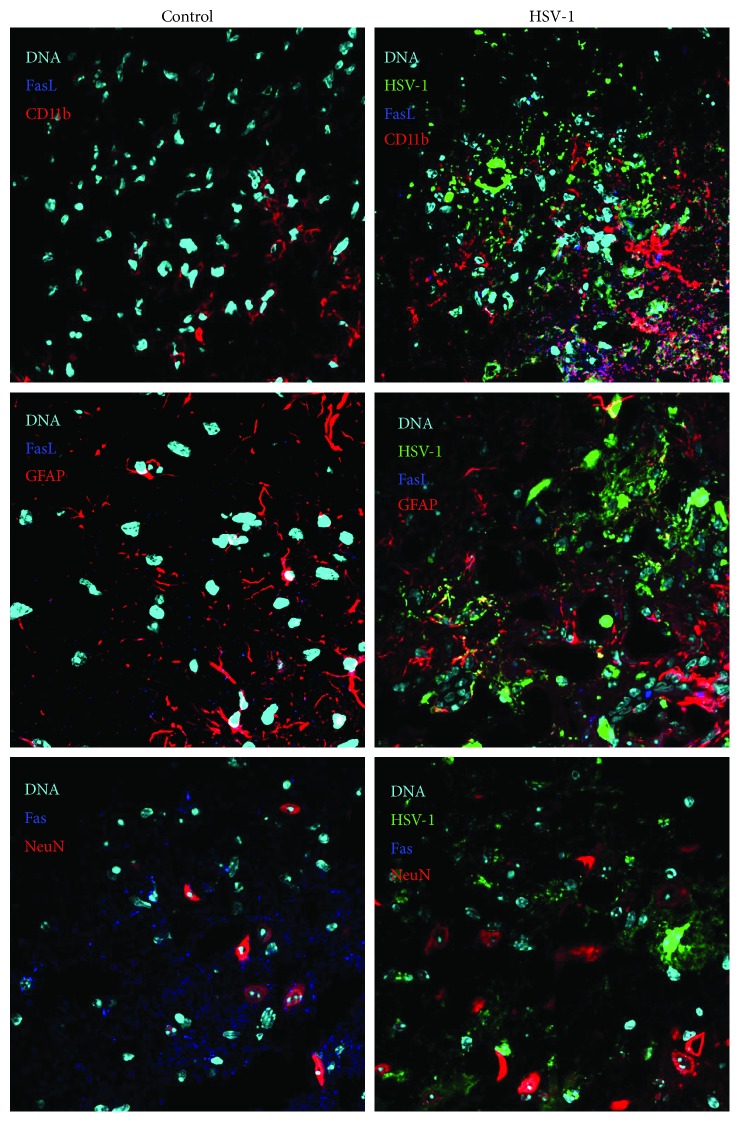
Microglia are the main source of FasL in HSV-1-infected brains. Representative confocal images of the brains obtained at day 5 p.i. with HSV-1. Tissue sections from brain stems were stained for microglia (CD11b+), astrocytes (GFAP+), neurons (NeuN), Fas, and FasL. Nuclei were counterstained with DAPI.

In the brains of HSV-1-infected BALB/c mice, FasL-positive cells were identified mostly as infected and uninfected CD11b+ cells surrounding HSV-1-infected sites, while only single FasL-positive GFAP+ cells were identified within the infected sites (Figure 6). Fas expression was detected in uninfected controls, while very little or no Fas expression was detected in HSV-1-infected brains (Figure 6). While we could detect cells positive for Fas expression in the uninfected control animals around neuronal cells, the infected area showed decreased Fas expression on the surrounding cells (Figure 6).

### 3.5. Nitric Oxide Production during HSV-1 Infection Adds to A*β* Accumulation

**Figure 7:**
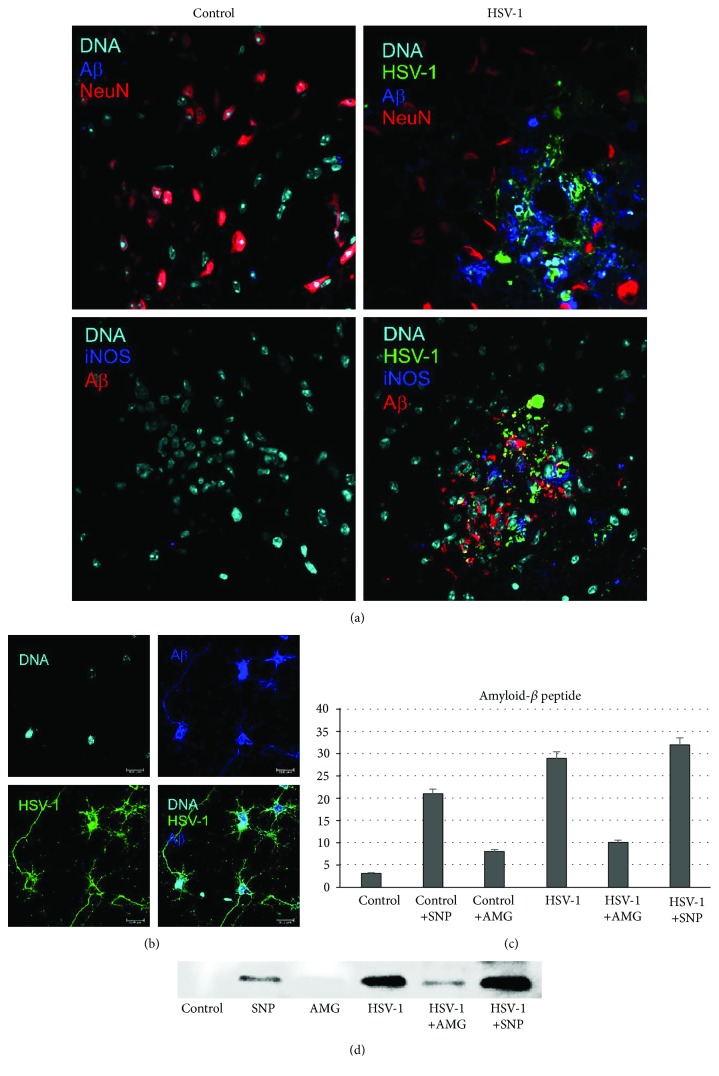
Nitric oxide production during HSV-1 infection adds to A*β* accumulation. (a) Representative confocal images of brains obtained at day 5 p.i. with HSV-1, with the hypothalamus and midbrains stained for HSV-1, A*β*, NeuN (neuronal marker), and iNOS. (b) Neuronal culture positive for A*β* and HSV-1. (c, d) Western blot analysis of A*β* in HSV-1-infected and control uninfected neuronal cultures subjected to treatment with the NO donor SNP (100 *μ*M) or the inhibitor of iNOS AMG (50 *μ*M) for 24 h. Means are expressed as the mean densitometric value ± SEM for *n* = 3. ^∗^Significant differences with *p* ≤ 0.05. Nuclei were counterstained with DAPI.

We performed tests to determine whether HSV-1 infection and NO production modulate amyloid precursor protein (APP) proteolysis processing into A*β*. It has been previously shown that HSV-1 causes the accumulation of intracellular A*β* in human neuroblastoma cells [6]. As a first step, the A*β* presence in cultured neurons and in the brains of HSV-1-infected mice was examined by immunofluorescence (Figures 7(a) and 7(b)). Intracellular or extracellular A*β* was barely detectable in uninfected cells. In contrast, HSV-1 infection induced a strong elevation of A*β* at 24 h p.i., both in neuronal culture and in the brains of HSV-1-infected mice (Figures 7(a) and 7(b)). A*β* was accumulated extracellularly around the HSV-1-infected neurons in the brains of HSV-1-infected mice at 5 d p.i. within the midbrain, brain stem, and hypothalamus (Figure 7(a)). A*β* also colocalized with HSV-1 antigens (Figures 7(a) and 7(b)). In primary neuronal cultures, A*β* was found mostly in the perikaryons of infected cells but also colocalized with HSV-1 antigens within processes (Figure 7(b)). In addition, we demonstrated colocalization of iNOS-positive cells at HSV-1-infected sites with accumulated A*β* (Figure 7(a)).

To confirm that not only HSV-1 infection but also NO production led to significant upregulation of A*β* levels, we incubated cultured neurons with the NO donor—SNP (100 *μ*M)—or the inhibitor of iNOS—AMG (50 *μ*M). Using Western blot analysis, we found that the NO donor significantly upregulated A*β* levels in both noninfected (positive control with NO donor) and HSV-1-infected neurons, in comparison to negative control (without NO donor) (Figures 7(c) and 7(d)). Furthermore, treatment with the inhibitor of iNOS caused downregulation of A*β* levels in infected neurons (Figures 7(c) and 7(d)).

## 4. Discussion

There is a growing body of evidence that nitric oxide (NO), a ubiquitous gaseous cellular messenger, plays significant roles in a variety of neurobiological processes. Several functions of this regulatory molecule have been identified in the nervous system, such as vasodilatation, neurotransmission, and host defence mechanisms [31, 32]. However, NO can exert both cytoprotective and cytotoxic effects in the central nervous system (CNS) [33, 34], depending on its concentration, time course exposure, and presence/absence of ROS at particular levels and cells. NO is a neuroprotectant at low levels, while it might behave as a toxicant at higher concentrations [35]. Overproduction of NO is caused by inducible NO synthase (iNOS), which is usually expressed by inflammatory phagocytic cells and other types of cells (e.g., epithelial and glial cells), and it has a defence function against bacteria, fungi, and parasites [36]. iNOS produces a much larger amounts of NO for a longer time than the other two constitutive enzymes, neuronal NOS and endothelial NOS [37].

NOS is induced in a variety of experimental virus infections in rats and mice, including Borna disease virus, herpes simplex virus type 1, and rabies virus, and in human diseases caused by human immunodeficiency virus-1 (HIV-1) and hepatitis B virus (HBV) [17, 18, 38, 39]. Furthermore, its induction in virus infection can be mediated indirectly by proinflammatory cytokines such as interferon-*γ* (IFN-*γ*) [40] and directly, by virus components, for example, an envelope glycoprotein of HIV, gp41, triggers iNOS expression in human astrocytes and murine cortical brain cells in culture [41]. Evidence suggests that localized production of NO early during HSV brain infection may be responsible for decreased neuronal infection [42, 43]. In this study, we found that antiviral effects of NO actually depended upon the type of HSV-1-infected cells and the concentration of the NO donor. In primary neuronal cultures, NO caused significant reduction of HSV-1 replication irrespectively of the used concentration, while in astrocytes, the NO donor supported HSV-1 replication if used at ≤100 *μ*M, while at higher concentrations, it blocked HSV-1 replication. Inhibition of iNOS activity in HSV-1-infected BALB/c mice led to upregulation of virus replication in trigeminal ganglia, but it had an opposite effect upon the brain infection. The results obtained here for trigeminal ganglia are consistent with papers published by Gamba et al. and Benencia et al., showing that the iNOS inhibitor, aminoguanidine, increased HSV-1 replication upon ocular and intranasal infection outside the CNS, respectively [42, 43]. The main sources of NO in HSV-infected trigeminal ganglia are dendritic cells (DCs) and monocytes/macrophages (Mo/M*ϕ*), which, together with type I IFNs, are essential for the early immune response against HSV [44]. Therefore, we may conclude that high levels of NO produced by macrophages and dendritic cells can block early stages of HSV infection in the peripheral nervous system, while low concentrations of NO actually provide advantageous effect for HSV replication in astrocytes. This may explain why the iNOS inhibitor—AMG—blocked replication of the virus in the brain later during infection.

There are reports showing that treatment of infected animals with the nonselective NOS inhibitor L-NMMA resulted in significantly improved survival rates, despite no decrease in viral titers [19, 45]. Similarly, experimental inhibition of NO during neuronal infection with West Nile virus (WNV) attenuated disease and prolonged survival [46]. During WNV encephalitis, more than 70% of the inflammatory monocyte-derived macrophages in the WNV-infected brain produced NO. Importantly, whereas inhibition of NO with AMG during the entire course of infection had no effect on the disease outcome, late inhibition resulted in enhanced survival of WNV-infected mice [46].

The main sources of NO in the brain during herpes encephalitis are microglia, as shown by Marques et al. [47]. In this study, we identified microglia and only a small portion of astrocytes *in vitro* as the main sources of NO in HSV-1-infected primary mixed glial cultures. Similarly, the brains of HSV-1-infected BALB/c mice indicated microglia as the main NO producers, rather than astrocytes, as shown previously [47].

Upon HSV-1 infection, microglia undergo activation, which is necessary for host defence, and are the source of proinflammatory cytokines and chemokines, such as tumor necrosis factor- (TNF-) *α*, interleukin- (IL-) 1*β*, CXCL10 [48], and NO [47]. However, the activated state of this cell type has also been linked to neurotoxicity, neurodegeneration, and chronic neuroinflammation in several disorders including Alzheimer's disease, Parkinson's disease, amyotrophic lateral sclerosis, and HIV-associated dementia [9, 10, 15]. Here, we found that NO influenced production of proinflammatory cytokines by primary neuronal cultures and mixed glial cultures.

In neuronal cultures, infection with HSV-1 upregulated IFN-alpha, TNF-alpha, CXCL9, and CXCL10, while addition of NO downregulated all tested cytokines and chemokines. Upon stress, neurons can release multiple cytokines and chemokines: interleukin 3 (IL-3), TNF-alpha, CXCL9, VEGF, L-selectin, IL-4, GM-CSF, IL-10, IL-1Ra, MIP, and CCL20 [49]. CXC-type chemokines, including CXCL9 and CXCL10, are potent chemoattractants for activated T cells, NK cells, monocytes, dendritic cells, and B cells [49, 50], while type I interferons (IFN-alpha and IFN-beta) are important in limiting viral replication and spread *in vitro*, but also *in vivo* at the periphery during initial HSV infection and virus reactivation [51]. Therefore, while NO produced by microglial cells helps to reduce viral replication in the infected neurons but not in astrocytes, it also downregulates cytokines important for antiviral response in the neuronal microenvironment. At the same time, NO may induce production of inflammatory TNF-alpha cytokine in the local site of HSV-1 infection. Therefore, prolonged or strong local production of NO may further add to HSV-1 local spread and further pathology by limiting the specific antiviral immune response. It has been previously suggested that NO affects the polarized Th1/Th2 response by a suppressive effect on Th1 subpopulations [52].

Upon HSV-1 infection, microglial cells undergo an abortive infection and induce a burst of proinflammatory cytokine and chemokine production. Following the HSV-1 infection, microglia also undergo a cell death—apoptosis and necrosis, while HSV-1-infected astrocytes suppress apoptosis. Here, we found that the NO donor at low levels protected HSV-1-infected microglia from secondary necrosis but it had no influence upon apoptosis. Taking into account that secondary necrotic cells are actually apoptotic cells, which did not undergo phagocytosis in vitro and microglia undergo abortive infection, we may conclude that NO protected microglia in HSV-1-infected glial cultures from early apoptosis following the virus entry.

Antiapoptotic actions of low levels of NO are numerous, ranging from an immediate interference with proapoptotic signalling cascades to long-lasting effects based on expression of cell protective proteins and the ability of NO to block caspases by S-nitrosylation/S-nitrosation [53]. Thus, we may conclude that NO exerts a double-edged role during HSV-1 infection. While it inhibits HSV-1 replication in neurons, NO contributes to microglia- and astroglia-induced chronic neuroinflammation and virus replication.

The extrinsic apoptotic pathway is initiated by ligand binding to the cell surface death receptor (tumor necrosis factor receptor) superfamily (e.g., Fas/CD95, tumor necrosis factor, and tumor necrosis factor-related apoptosis-inducing ligand- (TRAIL-) R1 and TRAIL-R2) [54]. However, emerging evidence accumulates on Fas as a mediator of apoptosis-independent processes including proliferation, angiogenesis, fibrosis, and inflammation. Cullen et al. observed that Fas-induced apoptosis of HeLa cells was associated with the production of an array of cytokines and chemokines, including IL-6, IL-8, CXCL1, MCP-1, and GM-CSF. Fas-induced production of MCP-1 and IL-8 promoted chemotaxis of phagocytes toward apoptotic cells, suggesting that these factors serve as “find-me” signals [55]. Furthermore, Fas/FasL death receptors activate apoptosis-independent inflammatory or proliferative signalling via the prototypic proinflammatory transcription factor NF-*κ*B or the mitogen-activated protein kinase (MAPK) family of kinases [56]. Krzyzowska et al. have previously shown that the Fas/FasL-dependent apoptotic pathway was a crucial mechanism for elimination of the inflammatory cells present in the HSV-2-infected sites within the vaginal epithelium during herpes genitalis [57]. Furthermore, Fas/FasL-dependent apoptosis of monocytes led to development of the local chemokine and cytokine milieu, necessary for mounting proper antiviral response [23, 57]. HSV-2-infected monocytes upregulated FasL during the whole tested period of HSV-2 infection, but Fas expression was elevated only early during infection to later decrease [57].

**Figure 8:**
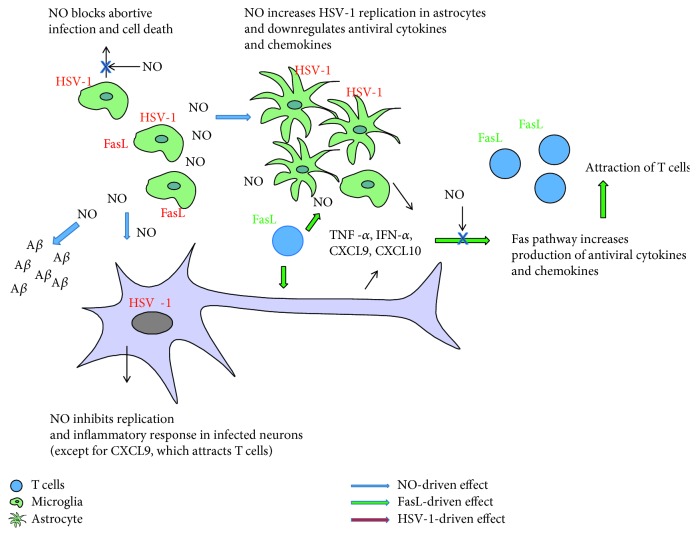
Schematic drawing depicting relations between HSV-1 infection, NO production, and the Fas/FasL pathway.

Here, we found that upon HSV-1 infection, both microglia and astrocytes were resistant to Fas-induced apoptosis. In addition, stimulation of Fas through soluble FasL led to significant upregulation of TNF-alpha and CXCL10 in uninfected and CXCL10 in HSV-1-infected neuronal cultures, while coaddition of the NO donor and sFasL reduced TNF-alpha and CXCL10 to the levels observed in untreated infected cultures. Only for CXCL9, coaddition of sFasL and the NO donor to HSV-1-infected culture showed a cumulative effect of a high expression level. The results obtained for CXCL9 follow those obtained previously for HSV-2-infected monocytes [57], which after Fas-stimulation upregulated both CXCL9 and TNF-alpha [57]. Similarly, as for HSV-2-infected mouse monocytes [57], HSV-1-infected microglia downregulate Fas expression and upregulate its ligand, FasL. Production of cytokines and chemokines together with NO through the Fas/FasL pathway has a primary role not only in reduction of replication and in attraction of cytotoxic T cells but also in reduction of inflammation. However, HSV-1 infection disturbs this natural antiviral mechanism—lack of Fas—or NO-induced cell death of microglia leads to excessive NO production and inflammatory reaction. Therefore, depending on the concentration, time course, and place, Fas/FasL together with NO can regulate a delicate balance between protection from HSV-1 neuroinfection and neuroinflammation (Figure 8).

Previously published studies have shown that both acute and latent HSV-1 brain infections are associated with oxidative damage [7, 9]. Recently, it has been shown that HSV infection-induced formation of reactive nitrogen and reactive oxygen species (RNS and ROS) leads to damage within the virus-infected brain. Ball [58] pointed out that the brain regions most frequently involved in herpes encephalitis are also the earliest and most severely involved targets of the neurodegenerative alterations of Alzheimer's disease (AD). A large prospective population-based study also showed that the risk of AD is increased in elderly subjects with positive titers of anti-HSV-1 IgM antibodies, which are markers of primary or reactivated HSV-1 infection [59]. One of the most widely accepted hypotheses on the molecular pathogenesis of AD focuses on the accumulation and aggregation of two proteins: A*β*, in the form of extracellular plaques, and hyperphosphorylated tau, as intracellular neurofibrillary tangles [11]. The accumulation of A*β* peptides in the extracellular spaces gives rise to the aggregates (plaques) that disrupt cell signalling, trigger inflammatory immune responses, and cause oxidative stress [60, 61]. Links between HSV-1 and AD include the discovery that the viral DNA is located very specifically within AD plaques [62] and that the main component of plaques, A*β*, accumulates in HSV-1-infected cell cultures [7] and in the brains of HSV1-infected mice [6]. Furthermore, A*β* is characterized by some degrees of sequence homology with HSV-1 glycoprotein B, so it may act as a seed for A*β* deposition in amyloid plaques [63]. Bourgade et al. showed that A*β* inhibited HSV-1 replication in fibroblast and epithelial and neuronal cell lines when added 2 h prior to or concomitantly with virus challenge, but not when added 2 or 6 h after virus challenge [64]. A*β* peptides also displayed antiviral activities against the enveloped influenza A virus [65]. Therefore, A*β* peptides can represent a novel class of antimicrobial peptides that protect against neurotropic virus infections such as HSV-1. It has been also suggested that antiviral treatments may open an antiviral approach for clinical therapy of AD [66].

Here, we also observed accumulation of A*β* both in HSV-1-infected neurons and in the extracellular space surrounding HSV-1-infected neurons *in vivo*. Furthermore, we also found that the NO donor increased accumulation of A*β* in uninfected primary neuronal cultures, while the iNOS inhibitor decreased its accumulation in HSV-1-infected neuronal cultures (Figure 8). The HSV-1-infected sites were surrounded by iNOS-positive CD11b cells. Several studies have suggested that NO modulates the processing of amyloid precursor protein (APP) and alters A*β* production [8, 9, 21]. Cai et al. [9] using human SH-SY5Y neuroblastoma cells stably transfected with wild-type APPwt695 demonstrated that low (physiological) levels of NO given in the form of sodium nitroprusside can inhibit the amyloidogenic processing of APP, whereas extra-high (pathological) concentrations of NO favor the amyloidogenic pathway of APP processing. Inflammatory and immune responses in the neuronal tissue involve increased iNOS activity in microglia and astrocytes, which further generates high levels of NO and peroxynitrite through the NO/superoxide pathway. Removal of iNOS in transgenic AD mice or the use of iNOS inhibitors to block NO production has been shown to protect against A*β*-induced neurotoxicity [67]. Accumulation of misfolded proteins such as A*β* is known to induce phosphorylation of eukaryotic initiation factor-2 (eIF2 *α*) [67]. Genetic and environmental risks for AD may influence modulation of the eIF2 *α* phosphorylation pathway. eIF2 *α* phosphorylation suppresses general protein synthesis, but it also induces translational activation of *β*-site APP cleaving enzyme 1 (BACE1), responsible for A*β* production [68]. NO can upstream induce and downstream mediate the kinases that phosphorylate eIF2*α*. Therefore, production of NO can indirectly add to activation of BACE1, responsible for development of amyloid plaques. In addition to being an upstream regulator of protein kinase R (PKR), NO production is regulated downstream by PKR [69]. It was also found that PKR activation was required for dsRNA-induced NF-*κ*B activation and iNOS expression in U373 MG astroglial cells [70].

## 5. Conclusion

This study may provide further evidence to clarify the molecular roles of NO and NO-related signalling during herpes simplex-induced neuroinflammation and neurodegeneration. It also indicates for the first time the existence of a link between Fas signalling due to neuroinflammation and nitrosative stress during HSV-1 infection. Further studies can contribute to finding of the potential molecular targets for a treatment of virus-induced neurodegeneration and A*β* accumulation.

## Figures and Tables

**Table 1:** Cytokine and chemokine expression in neuronal primary cultures.

	TNF-alpha	IFN-alpha	CXCL9	CXCL10
Control + NO donor	n.d.	n.d.	n.d.	n.d.
Control + iNOS inhibitor	n.d.	n.d.	n.d.	n.d.
sFasL	3.37 ± 0.8	n.d.	n.d.	52.22 ± 6.7
HSV-1	30.225 ± 5.1	24.25 ± 4.5	0.29 ± 0.2	109.13 ± 21.3
HSV − 1 + NO donor	10.69 ± 3.4^∗^	2.78 ± 0.9^∗^	n.d.	17.26±5.6^∗∗^
HSV − 1 + iNOS inhibitor	n.d.	n.d.	n.d.	n.d.
HSV − 1 + sFasL	8.59 ± 1.11^∗^	n.d.	n.d.	137.93 ± 3.1^∗^
HSV − 1 + NO donor + sFasL	3.86±0.9^∗∗^	n.d.	3.1^∗^ ± 0.5	53.2 ± 3.33^∗^

Cytokine and chemokine expression changes in the neuronal cultures at 24 h p.i. with HSV-1. Neuronal cultures were subjected to treatment with the NO donor—SNP (100 *μ*M)—and the inhibitor of iNOS—AMG (50 *μ*M)—and sFasL (0.1 *μ*g/ml). mRNA levels of IFN-alpha, TNF-alpha, CXCL9, and CXCL10 are shown as expression relative to control on the basis of the 2^−*∆∆*Ct^ method. mRNA levels were counted from three PCR reactions for each sample. ^∗^*p* ≤ 0.05 and ^∗∗^*p* ≤ 0.01 versus HSV-1-infected control. n.d. means not detected.

**Table 2:** Cytokine and chemokine expression in mixed glial primary cultures.

	TNF-alpha	IFN-alpha	CXCL9	CXCL10
Control + NO donor	0.71 ± 0.03	0.45 ± 0.01	0.94 ± 0.02	1.27 ± 0.34
Control + iNOS inhibitor	1.01 ± 0.02	1.1 ± 0.45	0.17 ± 0.04	1.1 ± 0.03
sFasL	8.88 ± 1.89^†^	4.5 ± 0.7^†^	45.2 ± 0.54^††^	n.d.
HSV-1	86 ± 9.1^††^	7422 ± 892^††^	152 ± 29^††^	424 ± 39^††^
HSV − 1 + NO donor	189±35^∗∗^	1247±299^∗∗^	95 ± 23^∗^	146±49^∗∗^
HSV − 1 + iNOS inhibitor	79 ± 12	10401±501^∗∗^	294±51^∗∗^	215±61^∗∗^
HSV − 1 + sFasL	43 ± 5^∗^	5673 ± 1329	445±91^∗∗^	552 ± 59^∗∗^
HSV − 1 + NO donor + sFasL	49 ± 6.6^∗^	6427 ± 987	110 ± 23^∗^	150±45^∗∗^

Cytokine and chemokine expression changes in the mixed glial cultures at 24 h p.i. with HSV-1. Mixed glial cultures were subjected to treatment with the NO donor—SNP (100 *μ*M)—or the inhibitor of iNOS—AMG (50 *μ*M). mRNA levels of IFN-alpha, TNF-alpha, CXCL9, and CXCL10 are shown as expression relative to control on the basis of the 2^−*∆∆*Ct^ method. mRNA levels were counted from three PCR reactions for each sample. ^∗^*p* ≤ 0.05 and ^∗∗^*p* ≤ 0.01 versus HSV-1-infected control and ^†^*p* ≤ 0.05 and ^††^*p* ≤ 0.01 versus uninfected control. n.d. means not detected.

## Data Availability

The data used to support the findings of this study are available from the corresponding author upon request.
